# Defining the Pros and Cons of Open, Conventional Laparoscopy, and Robot-Assisted Pyeloplasty in a Developing Nation

**DOI:** 10.1155/2014/850156

**Published:** 2014-02-02

**Authors:** Mrinal Pahwa, Archna R. Pahwa, Mohit Girotra, Rtika Ryfka Abrahm, Sachin Kathuria, Ajay Sharma

**Affiliations:** ^1^Sir Ganga Ram Hospital, 78-c, Mianwali Colony, Gurgaon 122001, India; ^2^Lady Hardinge Medical College, New Delhi 110001, India; ^3^University of Arkansas for Medical Sciences, Little Rock, AR 72205, USA

## Abstract

*Introduction*. Congenital pelviureteric junction obstruction (PUJO) is one of the most common causes of hydronephrosis. Historically, open dismembered pyeloplasty has been considered the gold standard intervention for correcting PUJO. The aim of this study was to compare the surgical and functional outcomes of three different approaches, namely, open, conventional laparoscopy, and robotic pyeloplasty. *Material and Methods*. 60 patients underwent minimally invasive pyeloplasty (30 conventional laparoscopies and 30 robotics) for congenital PUJO at a tertiary health center in India. Demographic, perioperative, and postoperative data were prospectively collected and analyzed. The data of these patients were retrospectively compared with another cohort of 30 patients who had undergone open pyeloplasty. *Results*. There was significant difference in operative time, time to drain removal, hospital stay, pain score, and complications rate between open and minimally invasive pyeloplasty (*P* < 0.05). SFI was considerably lesser in robotic as compared to conventional laparoscopy. The success rate in OP, CLP, and RP was 93.33, 96.67, and 96.67%. *Conclusion*. Robotic pyeloplasty is safe, effective, and feasible. It is associated with significantly lesser operative time, lesser blood loss, less pain, shorter hospital stay, and fewer complications. It is also associated with considerably lesser surgeon fatigue as compared to conventional laparoscopy pyeloplasty.

## 1. Introduction

Congenital pelviureteric junction obstruction (PUJO) is one of the most common causes of hydronephrosis. The cause of obstruction can be intrinsic, extrinsic, or both. Traditionally, open dismembered pyeloplasty (OP) has been considered the gold standard but it has its own drawbacks in terms of postoperative morbidity and poor cosmesis [1]. With the introduction of laparoscopy, it became possible to overcome these disadvantages and offer a minimally invasive approach. Conventional laparoscopic pyeloplasty (CLP) has success rate equivalent to open pyeloplasty with advantages of improved convalescence and lower morbidity [2, 3]. But CLP has its own limitations in the form of prolonged learning curve, advanced laparoscopy skills like intracorporeal suturing, surgeons fatigue, and increased operative time. Recent advances in robotic surgery have combined the advantages of open and laparoscopic surgery. We hereby undertook a study to compare the three approaches in terms of functional and postoperative outcomes.

## 2. Materials and Methods

We undertook a combined prospective and retrospective study in patients with primary PUJO. Sixty patients underwent minimally invasive pyeloplasty in the last two years by the same surgeon. The operating surgeon was already versed in laparoscopic pyeloplasty and had been performing these surgeries for the past 10 years. 30 patients underwent CLP and thirty patients underwent robot-assisted laparoscopic pyeloplasty (RP). Informed consent was taken from all the patients for the surgery and for recruitment in the study. Ethical clearance was obtained from the institute's ethical committee. The decision to choose either modality was based on patient's discretion, financial constraints, and availability of robot. Demographic, intraoperative and postoperative data was collected and analyzed. A surgeon fatigue index (SFI) was calculated according to the numerological rating scale. It is not a validated tool but just a rough estimate of surgeon's fatigue. The operating surgeon was asked to rate the discomfort after completion of the surgery on a scale of 1 to 10. Visual analog scale (VAS) was used to assess the pain in the postoperative period. Pain score was obtained once daily till the patients got discharged from the hospital before the administration of analgesics. Furthermore, thirty patients who had undergone open pyeloplasty by the same surgeon in the last 10 years were retrospectively studied and compared with the minimally invasive cohort. Patients had to be taken from the last ten years in open pyeloplasty group as only a few patients undergo open pyeloplasty after the advent and introduction of laparoscopic pyeloplasty at our institute. The diagnosis of primary PUJO was based on history and appropriate imaging modalities like ultrasound, intravenous pyelogram or contrast enhanced CT KUB, and nuclear renogram. Patients with prior abdominal surgery, incidentally detected PUJO, previous failed attempt at PUJO repair (endoscopic, open, or minimally invasive), bleeding diathesis, and active urinary tract infection were excluded from the analysis. Patients who underwent nondismembered pyeloplasty have also been excluded from this study. JJ stent was inserted intraoperatively in an antegrade manner. Stent was removed four weeks after surgery. Patient was initially followed up 10 days after surgery and then 6 weeks after stent removal followed by six months for one year and annually thereafter. The procedure was considered successful only if patients were subjectively relieved of their symptoms along with objective determination of unimpeded drainage on imaging studies. Patients were asked about their symptoms and imaging was limited to nuclear renography on each follow-up visit after stent removal. Statistical analysis was performed by the SPSS program for Windows, version 17.0. Continuous variables are presented as mean, and categorical variables are presented as absolute numbers and percentage. Continuous variables were compared using ANOVA. If the *F* value was significant and variance was homogeneous, Tukey multiple comparison test was used to assess the differences between the individual groups; otherwise, Tamhane's T2 test was used. Categorical variables were analyzed using the chi-square test. For all statistical tests, a *P* value less than 0.05 was taken to indicate a significant difference.

## 3. Our Technique

### 3.1. Minimally Invasive Pyeloplasty

Patient is placed in a modified flank position and secured to the operating table. The DaVinci robot is docked from the back of the patient with axis of the robot perpendicular to that of patient. We use a four port transperitoneal technique. Pneumoperitoneum is made by veeres needle at a point 4 cm above and lateral to umbilicus. 12 mm port is inserted through the same site. Two other 8 mm ports are inserted on either side of this port in midclavicular line with a minimum of 8 cm distance between either ports. An assistant 10 mm port is made in the midline 8 cm from the camera port. The operative technique is pretty much the standard. The colon is mobilized along the line of Toldt and reflected medially. Ureters are identified and traced till PUJ where it is transected. Anderson Hynes dismembered pyeloplasty was performed in most of the cases. After spatulating the ureter, ureteropelvic anastomosis is done with 4–0 vicryl in continuous manner. Antegrade 6 Fr JJ stent is inserted after completing posterior layer and its position checked by fluoroscopy. A perinephric drain is inserted and the trocars are removed under vision. The Foleys catheter is removed after two days and drain is removed when drain output is less than 50 mL per day. The stent is removed after 4 weeks and patient is followed up at 6-month intervals for one year. DTPA scan is performed at 6 months and one year later. Similar technique is followed while performing CLP.

### 3.2. Open Pyeloplasty

All patients underwent Anderson Hynes dismembered pyeloplasty by retroperitoneal approach through intercostal incision. The rest of the technique and follow up are the same as described above in minimally invasive pyeloplasty.

## 4. Results 

Table 1 shows the demographic and clinical profile of the patients. Patients were divided into three groups according to the surgery performed. All the groups were evenly matched and comparable in baseline parameters.  Table 2 shows the perioperative and postoperative outcomes in the three groups along with surgeon fatigue index.

The mean operative times were considerably lesser in OP and RP as compared to CLP. The mean difference in operative time between open and CLP was 64.2 minutes whereas it was 72.8 minutes while comparing CLP and RP, the difference being statistically significant using post hoc tests. Mean blood loss in OP, CLP and RP was 114.47, 55.24, and 46.37 mL. OP was associated with greater blood loss and the difference was statistically significant from CLP, and RP (*P* < 0.001). Drain removal was found to be the earliest in RP followed by CLP and OP. It was considerably shorter in patients with RP as compared to CLP and OP (*P* < 0.001). The hospital stay was also seen to be the shortest in RP cohort and CLP as compared to patients in OP group. (2.45 days in RP versus 3 days in CLP and 2.45 versus 4.83 days in OP, *P* < 0.001). As expected and routinely observed in clinical practice, patients in OP experienced more pain as compared to minimally invasive pyeloplasty groups. Pain score was calculated daily till the patient was discharged from the hospital using numerical rating scale. It was 7.9 in OP group compared to 4.46 in minimally invasive pyeloplasty groups, the difference being statistically significant. It is well known in the literature that CLP is associated with increased surgeon fatigue and injuries. We found laparoscopy to be considerably tiring when compared to RP (surgeon fatigue index 6.8 versus 3.9, *P* < 0.01). The morbidity rate in the OP, RP, and CLP was 16.1%, 8%, and 11.4%, respectively. Although the complication rate was higher in open group, the difference was not statistically significant. All the complications in the minimally invasive group were Clavien grade 1 or 2. The main complications observed in the open group were wound infection, urinary tract infection (UTI), pleural effusion, ileus, anastomotic leak, and basal atelectasis, whereas the minimally invasive group had prolonged drain output, UTI, and gut injury. Fewer complication rates relate to the minimally invasive nature of CLP and RP, improved magnification, and better suturing capabilities due to improved mobility of instruments.

Speaking of success rate, all three groups had equal efficacy. Out of the 90 patients analyzed, only 4 patients developed recurrences. Three patients had documented obstruction on nuclear renogram in the follow-up period whereas one patient developed bothersome pain and only partial obstruction on nuclear renogram. Two patients were in the OP group and one each in RP and CLP group.

## 5. Discussion

Since Kuster reported the first successful open pyeloplasty in 1891 [4], the surgical correction of the PUJO has been approached in many ways. Anderson-Hynes dismembered pyeloplasty, first described in 1949, remains most popular today [4]. However, the significant incisional morbidity, along with the extended recovery period and the increased need for narcotic analgesia, has led to the development of various minimally invasive treatment modalities. Laparoscopy allows significant better cosmesis, lower blood loss, lower pain, and improved convalescence with equivalent efficacy. However it required advanced laparoscopic skills with a steep learning curve [5–7] and remains limited to specialized laparoscopic centers. The introduction of robotic technology to this complex reconstructive surgery reduces the limitations of laparoscopy. The system improves and magnifies the view (3D), eliminates tremor, and provides full mobility of robotic instruments (seven degrees of freedom compared with four in laparoscopic surgery). Nevertheless, disadvantages to robotic-assisted laparoscopic surgeries are no tactile feedback, increased setup time, and cost.

In our series, mean age was 38.8 (range 23–62) years. In analyzing the literature on adults, we found that average age was in the range of 31–81 years [8–16]. Crossing vessels are a common finding in patients with PUJO. Detection of abnormal vessels has been reported in 26–100% of cases [8–16].

Numerous case series have been published on robotic pyeloplasty since Gettman et al. first reported their case series on RP [17].  Table 3 shows a list of published series on RP with total number of patients greater than 25. Only 4 papers could be identified which had more than 50 patients. In 2004, Peschel et al. published their results on 49 patients with mean operating time 124 min, blood loss of 50 mL, complication rate of 4%, and a success rate of 100% [18]. In the largest multicenter study conducted by Mufarrij et al., 140 patients underwent RP at 3 centers [16]. The mean operative time was 217 minutes, blood loss was 50 mL, complication rate was 9.2%, hospital stay was 2.1 days, and a success rate of 95.7%. Schwenter published their results on 92 patients with a mean followup of 39.1 months and a success rate of 97%. The mean operating time in their study was 108 minutes, blood loss was less than 50 mL, complication rate was 3.3%, and hospital stay was 4.57 days [14]. Recently, Gupta et al. reported their experience of 86 patients with mean operating time of 121 min, blood loss of 45 mL, average hospital stay of 2.5 days, and with a success rate of 97% [19]. In our study, mean operative time of RP was 147.25, mean blood loss was 46.37, mean hospital stay was 2.45 days, mean time to drain removal was 2.03 days, complication rate was 10%, and success rate was 96.7%. The results were consistent with those reported in the literature.

In 2002, Gettman et al. retrospectively compared their initial 6 patients after RP with 6 age-matched CLP controls and concluded that operative times were improved with robotic assistance (140 min versus 235 min) as was suturing time (70 min versus 120 min). Hospital stay (4 days), estimated blood loss (50 mL), and complications (none) were equivalent between the 2 groups [17]. Braga et al. carried out a meta-analysis and systematic review of RP versus CLP [25]. They found eight studies where these two approaches were directly compared. Of the eight studies that evaluated operative time, three showed similar operative time for RAP and CLP [11, 26, 27], four indicated that RAP time was shorter than CLP [13, 17, 20, 28] (with a nonstatistically significant difference in one study [20]), and the only prospective study revealed that CLP operative time was significantly shorter than RAP. Meta-analysis of extractable data from five studies demonstrated a significantly shorter hospital stay after RAP compared with CLP (random effects model; WMD: −0.5 d; 95% CI: −0.6–0.4; *P* < 0.01). Meta-analysis of these eight studies showed that both procedures had similar complication rates (random effects model; OR: 0.7; 95% CI: 0.3–1.6; *P* = 0.40). Of the eight studies that evaluated this outcome, five revealed a 100% success rate for both approaches [11, 13, 17, 21, 28], two showed higher success rates with RAP than with CLP (100% versus 97% and 99% versus 97%) [26, 27], and one had a better success rate with CLP than with RAP (100% versus 97%) [20]. While comparing these two types of minimally invasive pyeloplasty we observed that there was no significant difference between the two in terms of blood loss, hospital stay, complication rate, and success rate. The operative time was considerably shorter in robotic pyeloplasty as compared to laparoscopic pyeloplasty. The difference in operative time further increases as the experience increases with robotic surgery as it leads to faster docking. The time to drain removal was also found to be significantly lower in RP as it offers improved vision, more mobility of instruments, and decreased fatigue. As rightly pointed out by Rajeev et al, there is considerable surgeon fatigue in laparoscopic pyeloplasty. The surgeon fatigue index was found to be considerably lesser in RP in our study.

Calvert et al. reviewed 49 laparoscopic pyeloplasty with 51 open pyeloplasty within an overlapping period of 3 years. Compared with open procedures, laparoscopic procedures were associated with a longer mean operating time (159 versus 91 min; *P* < 0.001), a shorter mean time to normal diet (38 versus 72 h; *P* < 0.001), and a similar mean hospital stay (5 days; *P* = 0.6). The operative complication rates were 17% for primary laparoscopic pyeloplasties and 24% for primary open pyeloplasties [29]. Umari et al. retrospectively compared 24 open pyeloplasty with 25 cases of laparoscopic pyeloplasty. Laparoscopic pyeloplasty was found to have longer mean operating time (274 versus 143 min) and a shorter mean hospital stay (9.9 versus 15.8 day) and the perioperative complication rates were 16.7% for laparoscopic pyeloplasties and 20% for open pyeloplasties. The success rates were 90.5% for laparoscopy and 90.9% for open surgery [30]. Boylu et al. did a prospective study to compare the surgical and functional outcomes of minimally invasive pyeloplasty including robotic and laparoscopic versus open pyeloplasty. They concluded that minimally invasive pyeloplasty has low morbidity, short length of stay, and less blood loss compared with open surgical repair [31]. Mei et al. did an excellent systemic review and meta-analysis on laparoscopic versus open pyeloplasty in children. The OP has significantly reduced operative time (weighted mean difference [WMD] = 59.00; 95% confidence interval [CI] = 41.15 to 76.85; *P* < 0.00001) and higher stent placement rate (odds ratio [OR] = 5.97; 95% CI = 3.17 to 11.26; *P* < 0.00001) compared with LP, whereas the duration of hospital stay was shorter in the LP group (WMD = −0.40; 95% CI = −0.77 to −0.03; *P* = 0.03). No difference was observed between LP and OP regarding complications (OR = 0.78; 95% CI = 0.46 to 1.34; *P* = 0.37) or success rate (OR = 1.76; 95% CI = 0.71 to 4.36; *P* = 0.22). Mean operative time, blood loss, hospital stay, and complication rate in OP and CLP groups in our study were 191.56, 127.5; 55.24, 114.47; 3,4.83; and 11.4%, 16.1%, respectively. Pain scores were more in OP than in CLP group, the difference being statistically significant [32].

Though not analyzed in this study, financial cost plays an important role in deciding the type of surgery especially in a developing nation like India. Cost of robotic surgery is significantly higher (almost double) than open or conventional laparoscopic pyeloplasty. In terms of financial cost and loss of working hours, laparoscopy seems to offer the best solution. The low cost of open pyeloplasty has to be balanced against the increased morbidity associated with it and increased time taken to return to daily activity. Another limitation of the study is the combined prospective and retrospective nature of the study. The fact that the open cases were done over a period of 10 years and the minimally invasive cases were done over a period of two years is a source of likely selection bias.

## 6. Conclusions 

To conclude, pyeloplasty has comparable efficacy and success rate irrespective of the approach used. OP and RP have comparable operative time whereas CLP has considerably increased operative time. Although blood loss was more in OP group but it was not significantly more than in CLP and RP. Complication rate and hospital stay were lesser in CLP and RP than in OP group. In developing countries, OP offers a cheaper alternative to patient with equivalent efficacy and decreased operative time but at the cost of increased morbidity, increased blood loss, more pain, and increased time to return to daily activity. Considering all the parameters, RP emerges as the most favorable approach if available in terms of decreased operating time, blood loss, hospital stay, pain control, and lesser surgeon fatigue.

## Figures and Tables

**Table 1:** Clinical profile of patients in three groups.

	OP	CLP	RP
Number of patients	30	30	30
Mean age (years)	31.4 (21–45)	34.4 (23–39)	32 (19–49)
Gender (M/F)	14/16	13/17	12/18
BMI	24.7 (19–29)	25.5 (20–30)	26.4 (19–30)
Laterality (R/L)	13/17	15/15	14/16

**Table 2:** Perioperative and postoperative outcomes in the three groups and the significance indifference between minimally invasive group and OP group. *P* < 0.05 was considered significant.

	OP	CLP	RP	Significance *b*/*w* minimally invasive and open group
Operative time (mins)	127.5 (112–168)	191.56 (145–276)	141.73 (110–235)	*P* < 0.001
Mean blood loss (mL)	114.47 (65–198)	55.24 (35–120)	46.37 (30–95)	*P* < 0.001
Crossing vessel (%)	26.67	33.3	33.3	NS
Surgeon fatigue index	Not calculated	6.8	3.9	*P* < 0.05
Pain score	7.9	4.77	4.16	*P* < 0.001
Time to drain removal (days)	3.58 (2–12)	2.68 (1–8)	2.03 (1–6)	*P* < 0.001
Mean hospital stay (days)	4.83 (3–8)	3 (2–6)	2.45 (2–6)	*P* < 0.001
Complications (%)	16.1	11.4	8	NS, *P* = 0.28
Followup (months)	35 (24–60)	18 (8–23)	13.5 (5–20)	
Recurrence	2/30	1/30	1/30	NS, *P* = 0.81

NS: not significant.

**Table 3:** Summary of case series on robotic pyeloplasty.

Author	Year	Type of study	Number	Mean age (years)	Mean operative time (minutes)	Blood loss (mL)	Complications (%)	Hospital stay (days)	Success (%)
Gettman et al. [17]	2002	CC	9	NA	138.8	50	11.1	4.7	100
Peschel et al. [18]	2004	R	49	NA	124	50	0	5.5	100
Siddiq et al. [8]	2005	R	26	34.5	245	69	11.5	2	100
Mendez-Torres et al. [9]	2005	R	32	31.2	300	52	11.5	2	100
Palese et al. [10]	2005	R	38	39.3	225.6	77.3	10.5	2.9	94.7
Bernie et al.* [11]	2005	CR	7/7	32	324	60	28.5	2.5	100
Patel [12]	2005	R	50	31	122	40	0	1.1	100
Weise and Winfield* [20]	2006	CR	31/14	26	271	100	6	2.1	97
Link et al.* [21]	2006	CR	10/10	46.5	173.8	NA	10	NA	100
Atug et al. [13]	2006	R	44	32.8	219.4	50	0	1.1	100
Schwentner et al. [14]	2007	R	92	35.1	108	<50	3.3	4.57	96.7
Yanke et al. [22]	2008	R	29	41.2	196	39	13.7	2.2	100
Erdeljan et al. [15]	2008	R	55	na	171	57	1.8	2.3	95
Gupta et al. [19]	2010	R	85	24.8	121	45	9.4	2.5	96.5
Mufarrij et al. [16]	2008	R	140	38.5	217	59.4	9.2	2.1	95.7
Erdeljan et al. [23]	2010	R	90	na	167.7	53.4	5.5	2.53	93
Bird et al.* [24]	2011	CR	98/74	39.6	189	>50	5	2	100

R: retrospect; CC: case controlled; CR: comparative retrospect.

*comparative series comparing RP and CLP.

## References

[1] O’Reilly PH, Brooman PJC, Mak S (2001). The long-term results of Anderson-Hynes pyeloplasty. *BJU International*.

[2] Bauer JJ, Bishoff JT, Moore RG, Chen RN, Iverson AJ, Kavoussi LR (1999). Laparoscopic versus open pyeloplasty: assessment of objective and subjective outcome. *Journal of Urology*.

[3] Soulié M, Thoulouzan M, Seguin P (2001). Retroperitoneal laparoscopic versus open pyeloplasty with a minimal incision: comparison of two surgical approaches. *Urology*.

[4] Murphy LJT, Murphy LJT (1972). The kidney. *The History of Urology*.

[5] Smith AD (1984). Percutaneous ureteral surgery and stenting. *Urology*.

[6] Inglis JA, Tolley DA (1986). Ureteroscopic pyelolysis for pelviureteric junction obstruction. *British Journal of Urology*.

[7] Yanke BV, Lallas CD, Pagnani C, McGinnis DE, Bagley DH (2008). The minimally invasive treatment of ureteropelvic junction obstruction: a review of our experience during the last decade. *Journal of Urology*.

[8] Siddiq FM, Leveillee RJ, Villicana P, Bird VG (2005). Computer-assisted laparoscopic pyeloplasty: university of Miami experience with the daVinci*™* surgical system. *Journal of Endourology*.

[9] Mendez-Torres F, Woods M, Thomas R (2005). Technical modifications for robot-assisted laparoscopic pyeloplasty. *Journal of Endourology*.

[10] Palese MA, Munver R, Phillips CK, Dinlenc C, Stifelman M, DelPizzo JJ (2005). Robot-assisted laparoscopic dismembered pyeloplasty. *Journal of the Society of Laparoendoscopic Surgeons*.

[11] Bernie JE, Venkatesh R, Brown J, Gardner TA, Sundaram CP (2005). Comparison of laparoscopic pyeloplasty with and without robotic assistance. *Journal of the Society of Laparoendoscopic Surgeons*.

[12] Patel V (2005). Robotic-assisted laparoscopic dismembered pyeloplasty. *Urology*.

[13] Atug F, Burgess SV, Castle EP, Thomas R (2006). Role of robotics in the management of secondary ureteropelvic junction obstruction. *International Journal of Clinical Practice*.

[14] Schwentner C, Pelzer A, Neururer R (2007). Robotic Anderson-Hynes pyeloplasty: 5-Year experience of one centre. *BJU International*.

[15] Erdeljan P, Caumartin Y, Warren J (2008). Robotic pieloplasty: long-term follow-up of first Canadian experience. *Canadian Urological Association Journal*.

[16] Mufarrij PW, Woods M, Shah OD (2008). Robotic dismembered pyeloplasty: a 6-year, multi-institutional experience. *Journal of Urology*.

[17] Gettman MT, Peschel R, Neururer R, Bartsch G, Rassweiler J (2002). A comparison of laparoscopic pyeloplasty performed with the davinci robotic system versus standard laparoscopic techniques: initial clinical results. *European Urology*.

[18] Peschel R, Neururer R, Bartsch G, Gettman MT (2004). Robotic pyeloplasty: technique and results. *Urologic Clinics of North America*.

[19] Gupta NP, Nayyar R, Hemal AK, Mukherjee S, Kumar R, Dogra PN (2010). Outcome analysis of robotic pyeloplasty: a large single-centre experience. *BJU International*.

[20] Weise ES, Winfield HN (2006). Robotic computer-assisted pyeloplasty versus conventional laparoscopic pyeloplasty. *Journal of Endourology*.

[21] Link RE, Bhayani SB, Kavoussi LR (2006). A prospective comparison of robotic and laparoscopic pyeloplasty. *Annals of Surgery*.

[22] Yanke BV, Lallas CD, Pagnani C, Bagley DH (2008). Robot-assisted laparoscopic pyeloplasty: technical considerations and outcomes. *Journal of Endourology*.

[23] Erdeljan P, Caumartin Y, Warren J (2010). Robot-assisted pyeloplasty: follow-up of first Canadian experience with comparison of outcomes between experienced and trainee surgeons. *Journal of Endourology*.

[24] Bird VG, Leveillee RJ, Eldefrawy A, Bracho J, Aziz MS (2011). Comparison of robot-assisted versus conventional laparoscopic transperitoneal pyeloplasty for patients with ureteropelvic junction obstruction: a single-center study. *Urology*.

[25] Braga LHP, Pace K, DeMaria J, Lorenzo AJ (2009). Systematic review and meta-analysis of robotic-assisted versus conventional laparoscopic pyeloplasty for patients with ureteropelvic junction obstruction: effect on operative time, length of hospital stay, postoperative complications, and success rate. *European Urology*.

[26] Franco I, Dyer LL, Zelkovic P (2007). Laparoscopic pyeloplasty in the pediatric patient: hand sewn anastomosis versus robotic assisted anastomosis-Is there a difference?. *Journal of Urology*.

[27] Kim S, Canter D, Leone N, Patel R, Casale P (2008). A comparative study between laparoscopic and robotically assisted pyeloplasty in the pediatric population. *Journal of Urology*.

[28] Bhayani SB, Link RE, Varkarakis JM, Kavoussi LR (2005). Complete daVinci*™* versus laparoscopic pyeloplasty: cost analysis. *Journal of Endourology*.

[29] Calvert RC, Morsy MM, Zelhof B, Rhodes M, Burgess NA (2008). Comparison of laparoscopic and open pyeloplasty in 100 patients with pelvi-ureteric junction obstruction. *Surgical Endoscopy and Other Interventional Techniques*.

[30] Umari P, Lissiani A, Trombetta C, Belgrano E (2011). Comparison of open and laparoscopic pyeloplasty in ureteropelvic junction obstruction surgery: report of 49 cases. *Archivio Italiano di Urologia e Andrologia*.

[31] Boylu U, Basatac C, Turan T, Onol FF, Gumus E (2012). Comparison of surgical and functional outcomes of minimally invasive and open pyeloplasty. *Journal of Laparoendoscopic & Advanced Surgical Techniques A*.

[32] Mei H, Pu J, Yang C, Zhang H, Zheng L, Tong Q (2011). Laparoscopic versus open pyeloplasty for ureteropelvic junction obstruction in children: a systematic review and meta-analysis. *Journal of Endourology*.

